# Knowledge of the ovulatory cycle and its determinants among women of reproductive age in Papua New Guinea: Insights from a population-based study

**DOI:** 10.1371/journal.pone.0324255

**Published:** 2025-05-28

**Authors:** McKenzie Maviso, Gracelyn Potjepat, Elias Namosha, Nancy Geregl, Paula Z. Aines, Lisa M. Vallely

**Affiliations:** 1 Division of Public Health, School of Medicine and Health Sciences, University of Papua New Guinea, Port Moresby, Papua New Guinea; 2 School of Social Sciences, Faculty of Arts, Social Sciences & Humanities, University of Wollongong, Northfields Ave Wollongong, Australia; 3 Division of Nursing, School of Medicine and Health Sciences, University of Papua New Guinea, Port Moresby, Papua New Guinea; 4 School of Health Sciences, Pacific Adventist University, Port Moresby, Papua New Guinea; 5 Papua New Guinea Institute of Medical Research, Goroka, Eastern Highlands Province, Papua New Guinea; 6 Asia and Pacific Health Program, The Kirby Institute, UNSW Sydney, Kensington, Australia; University of Salamanca, SPAIN

## Abstract

**Background:**

Correct knowledge of the ovulatory cycle is crucial for preventing unintended pregnancies and improving women’s reproductive health. However, the factors affecting this knowledge among women in Papua New Guinea (PNG) remain unclear. This study aimed to assess the prevalence and determine the factors influencing women’s knowledge of the ovulatory cycle in PNG.

**Methods:**

Data from the PNG Demographic and Health Survey (DHS) was analyzed. Multivariable logistic regressions were used to determine factors associated with women’s knowledge of the ovulatory cycle. Adjusted odds ratios (aOR) with their 95% Confidence Intervals (CI) were reported. A p ≤ 0.05 was considered statistically significant.

**Results:**

Of 12,580 women in this study sample, 22% (n = 2,773) had correct knowledge of the ovulatory cycle. Women from the Highlands region (aOR 1.31, 95% CI: 1.00–1.88) and the Momase region (aOR 1.56, 95% CI: 1.15–2.13), those who identified as Christians (aOR 3.01, 95% CI: 1.38–6.59), owned a mobile phone (aOR 1.29, 95% CI: 1.04–1.59), read a newspaper or magazine (aOR 1.30, 95% CI: 1.10–1.54), and had Internet access (aOR 1.21, 95% CI: 1.00–1.85) had higher odds of correct knowledge of the ovulatory cycle. Similarly, those who knew any contraceptive method (aOR 2.13, 95% CI: 1.58–2.87) and currently used the modern method (aOR 1.26, 95% CI: 1.02–1.56) or traditional/folkloric method (aOR 1.82, 95% CI: 1.36–2.43) were more likely to have correct knowledge of the ovulatory cycle. However, knowledge of the ovulatory cycle remained lower among women aged 15–24 and 25–34, those with lower education levels, and those from the Southern region.

**Conclusions:**

In this study, less than a quarter of women had correct knowledge of the ovulatory cycle. Promoting reproductive health knowledge and awareness through educational curricula and mass media platforms could enhance women’s understanding of the ovulatory cycle, particularly among younger and less educated and empower them to make informed decisions about their reproductive health.

## Introduction

Ovulation in women of reproductive age is a natural physiological process and an indicator of fertility [1]. It refers to the phenomenon of the rupture of a fully mature ovarian follicle to release its content, the female gamete known as ova or egg into the fallopian tube for fertilization [1,2]. The ovulatory cycle involves three phases: follicular egg formation, ovulation, and luteal egg release, with ovulation being the most crucial event in which a mature egg is released from the ovary into the fallopian tube in preparation for fertilization [1,3]. The time of ovulation is determined by basal body temperature and cervical mucus, which occurs at the midpoint of the menstrual cycle, typically 14 days after the onset of menstruation (i.e., when the period begins), for an average 28-day cycle [3,4].

Knowledge of the ovulatory cycle is the basis of natural family planning methods [5]. It is essential for the successful practice of intercourse-related methods such as periodic abstinence, and condom use [6,7], especially when sexually active women may have limited access to modern contraceptive methods [5,8]. An understanding of ovulation can assist in diagnosing certain pathophysiological and medical conditions, as well as reducing the likelihood of unintended pregnancies and unsafe abortions [8].

In low- and middle-income countries (LMICs), sexual and reproductive health knowledge among women of reproductive age, such as fertility awareness and the ovulatory cycle, tends to be limited [7,9,10]. For instance, in sub-Saharan Africa, only 8.3% of women knew about ovulation and fertility [10]. A cross-sectional study in India found that only 21.2% of women of reproductive age had ovulatory cycle knowledge [11]. Similar studies in Ethiopia and Haiti further revealed that 24% of women were knowledgeable about the ovulatory cycle [12–14]. Understanding the physiology of ovulation is crucial for women, both for planning conception and avoiding unintended pregnancies [12,13].

Similarly, incorrect knowledge of the ovulatory cycle, particularly in the absence of modern contraceptives, is likely to contribute to undesirable sexual and reproductive health outcomes, such as unintended pregnancies and unsafe abortions [10,15,16]. A study in the United States of America revealed that over half (54%) of women were unaware of their next menstruation, with 47% lacking knowledge about ovulation and 67% uncertain about its timing (67%) [8]. A cross-sectional study in sub-Saharan Africa found that incorrect knowledge of ovulation remained a significant predictor of unintended pregnancies and ranged between 9% and 60% in the Republic of Benin and Namibia, respectively [10]. Similarly, women who lacked correct knowledge of the fertile period and the ovulatory cycle were almost twice as likely to have unsafe abortions in Ghana [16].

Situated in the Western Pacific region, PNG has a low contraceptive uptake, and women experience substantial challenges concerning family planning services [17,18]. The contraceptive prevalence rate stands at 20%–24% (rural-urban), with an unmet need for contraception at 25% [19,20]. Arguably, access to family planning services and utilization of contraceptives are frequently understated in broader public health discussions, as is the unmet need for contraception, particularly for rural women in PNG [21,22]. A recent population-based survey among 2,345 women found that 74% of women were using a modern contraceptive method ranging from injectables (45%) to other modern methods (0.23%) in PNG [17]. More recently, Dadzie et al. [23] estimate that 33%, 19%, and 4% of the women in PNG had discontinued Depo Provera injection, oral contraceptive pills, and other methods of contraception. The low uptake and discontinuation rates of modern contraception are attributable to several factors, such as cultural norms and practices, socioeconomic status, and place of residence, opting for another baby, as well as a lack of knowledge and information on contraceptive options [17,21,23,24]. In the absence of any contraceptive use, understanding one’s ovulatory cycle among women of reproductive age can assist in planning conception, preventing unintended or unwanted pregnancies leading to improved maternal health outcomes.

Even though knowledge of the ovulatory cycle remains a fundamental aspect of female reproductive health, there is limited evidence from PNG. Previous studies found a significant association between socio-cultural norms and menstrual practices [25,26]. However, none of these studies considered factors associated with knowledge of the ovulatory cycle at the population level. Evaluating reproductive women’s knowledge of the fertility period and ovulatory cycle is critical in an era of increasing unmet needs for modern contraception. The current study aimed to assess the prevalence and determine the factors influencing knowledge of the ovulatory cycle among women of reproductive age (15–49 years) in PNG using nationally representative survey data.

## Methods

### Study setting

PNG is in the eastern half of the island of New Guinea and is positioned to the north of Australia. The country has an area of 462.84 km² with an estimated population of over 10 million, of which more than 80% live in rural areas [18]. Over half (51%) of the population are men aged 15–49 years, while 49% are women of reproductive age. The fertility rate is estimated at 4.2 children per woman; 12% of pregnancies occur in women aged 15–19 [27].

### Data source and study sample

The study utilized secondary data from the most recent PNG Demographic and Health Survey (DHS) collected from October 2016 to December 2018. Among other demographic information, the DHS team collected specific information on maternal and child health, such as fertility, contraceptive use, parity, breastfeeding and infant feeding practices, child’s nutritional status, immunization, women’s empowerment, intimate partner violence, awareness and behavior regarding HIV and other sexually transmitted infections, and other health-related factors. The Inner-City Fund (ICF), as part of the DHS Program, provided technical assistance for the survey. Financial support was provided by the PNG Government, the Department of Foreign Affairs and Trade (DFAT) of Australia, the United Nations Population Fund (UNFPA), and UNICEF [27]. The survey used the list of census units (CUs) from the 2011 National Population and Housing Census as the sampling frame.

The PNGDHS used a two-stage stratified sampling technique. In the first stage, 800 CUs were selected, which was achieved through probability proportional to CU size [27]. The second stage involved selecting 24 households from each cluster using equal probability systematic selection, resulting in a sample of about 19,200 households. During the survey, 18,175 women of reproductive age (15–49 years) were identified in the interviewed households for individual interviews, with 15,198 women completing the interviews, yielding a response rate of 84%. For this study, a total weighted sample of 12,580 individuals, all of whom provided complete information on the variables of interest, was included [27]. Details of the methodology, pretesting, training field workers, sampling design, and selection are available in the PNGDHS final report and are accessible via the following link: https://dhsprogram.com/publications/publication-FR364-DHS-Final-Reports.cfm.

### Study variables and measurements

#### Outcome variable.

The outcome variable of this study was having the correct knowledge of the ovulatory cycle. In the DHS, women were asked, “When is the ovulation time?” Responses were categorized as “during her period,” “after the period ended,” “middle of the cycle,” “before the period begins,” “at any time,” and “I don’t know.” The outcome variable was recoded and dichotomized. A woman who responded, “middle of the cycle” was considered to have correct knowledge of the ovulatory cycle and coded as “1.” All other responses were considered to have incorrect knowledge of the ovulatory cycle and were coded as “0”.

#### Explanatory variables.

The study considered explanatory variables based on evidence from previous studies [7,9,11,12,14]. Sociodemographic variables include age, marital status, education level, respondent currently working, wealth index, place of residence, region, and religion. Maternal health-related variables include having had menstruation in the last six weeks, knowledge of any contraceptive method, current contraceptive use by method or type, and recent sexual activity in the last four weeks. Additional explanatory variables include having read a newspaper/magazine, listening to the radio, watching television, and mobile phone ownership.

### Statistical analysis

Data from the individual women’s files were extracted and cleaned, and all missing observations were omitted for analysis. Only the respondents with completed cases of variables of interest were included in the final analysis. Women’s sample weight (v005/1,000,000) was applied in all analyses to alleviate biased estimates based on the DHS guidelines. Both the descriptive and inferential statistics were performed. Descriptive statistics were used to summarize the characteristics of the study participants. A bivariate analysis was performed using the Chi-square (χ^2^) test of independence to determine the association between the outcome variable and explanatory variables. Variables that were not statistically significant at an alpha level of 0.05 in the bivariate analysis were omitted from the multivariable logistic regression analysis. The Variance Inflation Factor (VIF) command was used to test multicollinearity between the independent variables. The results indicated no evidence of multicollinearity (Mean VIF = 1.29, maximum VIF = 1.83, minimum VIF = 1.00).

To ascertain determinants of knowledge of the ovulatory cycle, multivariable logistic regression analysis was employed. A complex sampling plan was implemented using the “csplan” file to adjust for data strata, clusters, and sample weights to account for the complex survey methodology and standard errors. The results were presented as adjusted Odds Ratios (aORs) with 95% Confidence Intervals (CIs). Statistical significance was set at p ≤ 0.05. All analyses were performed using IBM Statistical Package for the Social Sciences (SPSS) Version 30.0 for Windows (Armonk, NY: IBM Corp.).

### Ethics consideration

Ethics approval was not required for this study since the data is secondary and is available in the public domain. To access and analyze the dataset, official permission was obtained from the DHS Program (https://dhsprogram.com/). Written informed consent was obtained from the participants prior to each interview. The participants’ anonymity and confidentiality were assured. All methods were performed in accordance with the relevant guidelines and regulations.

## Results

### Characteristics of study participants

A total weighted sample of 12,580 women was included in the analysis. Over one-third (37.9%) were aged 15–24, with a mean age of 29.42 (SD ± 9.89), and nearly two-thirds (63.7%) were married. About half (50.3%) of the respondents had only attained primary education, and most were not working (69.8%). Most of the women lived in rural areas (86.3%), and a significant proportion were from the Highlands region (41%). In terms of media exposure, more than one-third of the women owned a mobile phone (35.5%), read a newspaper or magazine (43.6%), listened to the radio (36.8%), and about 25% watched the television. Regarding contraceptives, nearly all the women knew of a contraceptive method (83.7%). Less than a quarter (22%) of women reported to have used modern methods at the time of the survey (**Table 1**).

**Table 1:** Characteristics of study participants (N = 12,580).

Variables	Weighted frequency (%)
**Age (years)**	
15–24	4,763 (37.9)
25–34	3,599 (28.6)
35–44	3,068 (24.4)
45 or more	1,152 (9.1)
**Marital status**	
Never married	3,610 (28.7)
Married	8,015 (63.7)
Divorced/separated/widowed	955 (7.6)
**Education level**	
No education	2,863 (22.8)
Primary	6,325 (50.3)
Secondary	2,921 (23.2)
Higher	471 (3.7)
**Husband/partner education level (Missing, n = 4,733)**	
No education	1,393 (17.8)
Primary	3,355 (42.8)
Secondary	2,256 (28.7)
Higher	843 (10.7)
**Respondent currently working**	
Yes	3,793 (30.2)
No	8,787 (69.8)
**Wealth index**	
Poorest	2,259 (18.0)
Poorer	2,303 (18.3)
Middle	2,340 (18.6)
Richer	2,600 (20.7)
Richest	3,078 (24.5)
**Place of residence**	
Rural	10,861 (86.3)
Urban	1,719 (13.7)
**Region**	
Southern	2,357 (18.7)
Highlands	5,157 (41.0)
Momase	3,243 (25.8)
Islands	1,822 (14.5)
**Religion**	
Christian	12,406 (98.6)
Non-Christian/no religion	174 (1.4)
**Own a mobile phone**	
Yes	4,463 (35.5)
No	8,117 (64.5)
**Read newspaper/magazine**	
Yes	5,481 (43.6)
No	7,099 (56.4)
**Listen to radio**	
Yes	4,624 (36.8)
No	7,956 (63.2)
**Watch television**	
Yes	3,168 (25.2)
No	9,412 (74.8)
**Internet access**	
Yes	1,711 (13.6)
No	10,869 (86.4)
**Had menstruation (last 6 weeks)**	
Yes	10,137 (80.6)
No	2,443 (19.4)
**Knowledge of any contraceptive method** ^**±**^	
Yes	10,534 (83.7)
No	2,046 (16.3)
**The current contraceptive method used by type**	
Modern methods	2,769 (22.0)
Traditional/folkloric methods	516 (4.1)
No method	9,295 (73.9)
**Recent sexual activity (last 4 weeks)**	
Yes	9,840 (78.2)
No	2,740 (21.8)

^±^Any contraceptive method includes modern contraceptives (e.g., injectable, pills, implants, intra-uterine devices (IUD), condoms, and diaphragm) and sterilization (e.g., tubal ligation and vasectomy). Other methods include traditional (e.g., periodic abstinence, withdrawal, and lactational amenorrhea) and folkloric (e.g., rhythm method, coitus interruptus, beliefs, rituals, abdominal massage, and herbal remedies).

### Knowledge of the ovulatory cycle among women of reproductive age

**Fig 1:**
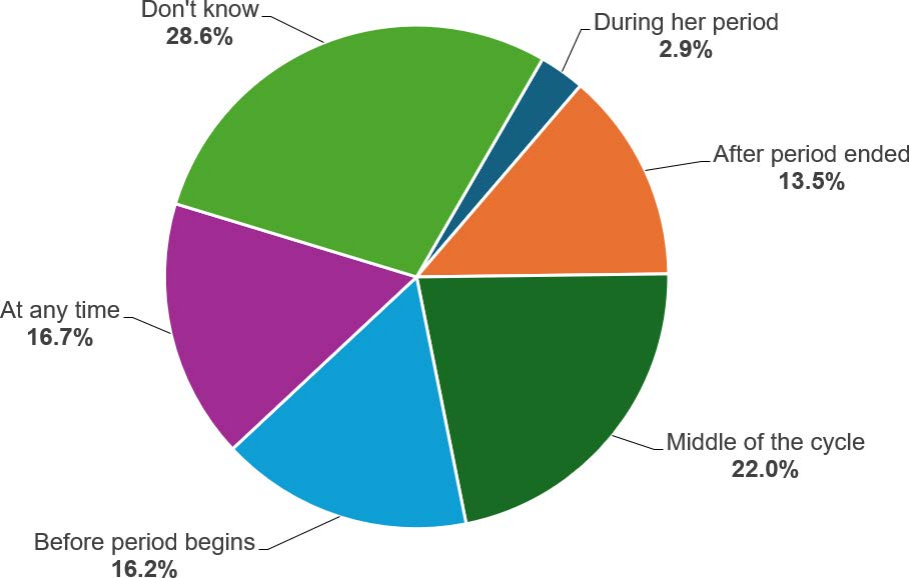
Distribution of knowledge of the ovulatory cycle among women of reproductive age in PNG.

Of the total weighted study sample, 22% (95% CI: 21.2–22.6%) had correct knowledge of the ovulatory cycle (i.e., middle of the cycle). In contrast, more than three-quarters (78%) of them had incorrectly responded to their understanding of the ovulatory cycle. Nearly one-third (28.6%) of women reported that they “don’t know” the timing of ovulation (**Fig 1**).

### Bivariate analysis of determinants of knowledge of the ovulatory cycle

Based on the bivariate analysis, correct knowledge of the ovulatory cycle was significantly low among younger women aged 15–24 (16.9%) compared to those aged 45 or older (28.6%). About 43% of women with higher education levels were knowledgeable about the ovulatory cycle, compared to those with lower education levels. In addition, correct knowledge of the ovulatory cycle was lower among women from the poorest wealth index (15.8%), living in rural areas (21%), and from the Southern region (18.3%). In terms of mass media exposure, women who read newspapers or magazines (23.1%), listened to the radio (26.2%), watched television (26.9%), and had Internet access (31.6%) reported having correct knowledge of the ovulatory cycle compared to those who did not. Similarly, a correct understanding of the ovulatory cycle was more common among women who knew about any contraceptive method (24.6%), and those who were using traditional or folkloric methods at the time of the survey (35.1%). In the Chi-square analysis, age, women’s education level, husband’s education level, wealth index, place of residence, region, mobile phone ownership, reading the newspaper or magazine, listening to the radio, watching television, Internet access, knowledge of any contraceptive method, and current contraceptive use by type were significantly associated with knowledge of the ovulatory cycle (p < 0.05). There is no statistically significant association found between having menstruation in the last six weeks and recent sexual activity and knowledge of the ovulatory cycle (**Table 2**).

**Table 2:** Bivariate analysis of determinants of knowledge of the ovulatory cycle (N = 12,580).

	Knowledge of the ovulatory cycle		
Variables	Incorrect (%)	Correct (%)	p-value^*^
**Sample**	9,807 (78.0)	2,773 (22.0)	
**Age (years)**			< 0.001
15–24	3,960 (83.1)	803 (16.9)	
25–34	2,751 (76.4)	848 (23.6)	
35–44	2,274 (74.1)	793 (25.9)	
45 or more	822 (71.4)	329 (28.6)	
**Marital status**			0.433
Never married	2,837 (78.6)	772 (21.4)	
Married	6,235 (77.8)	1,779 (22.2)	
Divorced/separated/widowed	734 (76.9)	221 (23.1)	
**Education level**			< 0.001
No education	2,384 (83.3)	479 (16.7)	
Primary	5,007 (79.2)	1,318 (20.8)	
Secondary	2,150 (73.6)	772 (26.4)	
Higher	267 (56.7)	204 (43.3)	
**Husband/partner education level (Missing, n = 4,733)**			0.001
No education	1,084 (77.8)	309 (22.2)	
Primary	2,660 (79.3)	695 (20.7)	
Secondary	1,748 (77.5)	508 (22.5)	
Higher	613 (72.7)	230 (27.3)	
**Respondent currently working**			0.125
Yes	2,925 (77.1)	869 (22.9)	
No	6,883 (78.3)	1,904 (21.7)	
**Wealth index**			< 0.001
Poorest	1,902 (84.2)	358 (15.8)	
Poorer	1,848 (80.3)	454 (19.7)	
Middle	1,833 (78.3)	508 (21.7)	
Richer	2,004 (77.1)	596 (22.9)	
Richest	2,221 (72.2)	857 (27.8)	
**Place of residence**			< 0.001
Rural	8,580 (79.0)	2,280 (21.0)	
Urban	1,227 (71.4)	493 (28.6)	
**Region**			< 0.001
Southern	1,925 (81.7)	432 (18.3)	
Highlands	4,093 (79.4)	1,064 (20.6)	
Momase	2,351 (72.5)	893 (27.5)	
Islands	1,438 (78.9)	384 (21.1)	
**Religion**			< 0.001
Christian	9,650 (77.8)	2,757 (22.2)	
Non-Christian	157 (90.8)	16 (9.2)	
**Own a mobile phone**			< 0.001
Yes	3,163 (70.9)	1,300 (29.1)	
No	6,644 (81.9)	1,473 (18.1)	
**Read newspaper or magazine**			0.013
Yes	4,212 (76.9)	1,269 (23.1)	
No	5,595 (78.8)	1,504 (21.2)	
**Listen to radio**			< 0.009
Yes	3,411 (73.8)	1,213 (26.2)	
No	6,396 (80.4)	1,560 (19.6)	
**Watch television**			< 0.001
Yes	2,317 (73.1)	852 (26.9)	
No	7,490 (79.6)	1,921 (20.4)	
**Internet access**			< 0.001
Yes	1,170 (68.4)	541 (31.6)	
No	8,637 (79.5)	2,232 (20.5)	
**Had menstruation (last 6 weeks)**			0.724
Yes	7,909 (78.0)	2,228 (22.0)	
No	1,898 (77.7)	545 (22.3)	
**Knowledge of any contraceptive method**			< 0.001
Yes	8,052 (75.4)	2,620 (24.6)	
No	1,904 (91.3)	182 (8.7)	
**Current type of contraceptive method used**			< 0.001
Modern methods	2,024 (73.1)	745 (26.9)	
Traditional/folkloric methods	335 (64.9)	181 (35.1)	
No method	7,448 (80.1)	1,847 (19.9)	
**Recent sexual activity (last 4 weeks)**			0.324
Yes	7,652 (77.8)	2,188 (22.2)	
No	2,155 (78.6)	585 (21.4)	

* Chi-square, p ≤ 0.05

### Determinants of correct knowledge of the ovulatory cycle

In the multivariable logistic regression analysis, age, education level, region, religion, mobile phone ownership, Internet access, knowing any contraceptive method, and the current type of method used were significantly associated with knowledge of the ovulatory cycle. The odds of correct knowledge of the ovulatory cycle remained higher among women from the Highlands region (aOR 1.31, 95% CI: 1.00–1.88, p < 0.001) and the Momase region (aOR 1.56, 95% CI: 1.15–2.13, p < 0.001), those who identified as Christians (aOR 3.01, 95% CI: 1.38–6.59, p = 0.006), owned a mobile phone (aOR 1.29, 95% CI: 1.04–1.59, p = 0.023), read a newspaper or magazine (aOR 1.30, 95% CI: 1.10–1.54, p = 0.002) and had Internet access (aOR 1.21, 95% CI: 1.00–1.85, p = 0.05). Similarly, women who knew any contraceptive method (aOR 2.13, 95% CI: 1.58–2.87, p < 0.001) and currently using modern methods (aOR 1.26, 95% CI: 1.02–1.56, p < 0.001) or traditional/folkloric methods (aOR 1.82, 95% CI: 1.36–2.43, p < 0.001) had a correct knowledge of the ovulatory cycle. However, women from the Southern region (aOR 0.87, 95% CI: 0.65–1.18, p < 0.001), aged 15–24 (aOR 0.55, 95% CI: 0.42–0.73, p < 0.001) and aged 25–34 (aOR 0.68, 95% CI: 0.52–0.89, p < 0.001), those who had no formal education (aOR 0.41, 95% CI: 0.21–0.78, p = 0.003), had primary education (aOR 0.53, 95% CI: 0.29–0.99, p = 0.003) and had secondary education (aOR 0.65, 95% CI: 0.31–1.36, p = 0.003), had lower odds of having correct knowledge of the ovulatory cycle (**Table 3**).

**Table 3:** Multivariable analysis of correct knowledge of the ovulatory cycle and its determinants.

Variables	Crude OR (95% CI)	Adjusted OR (95% CI)	P-value
**Age (years)**			0.000^**^
15–24	0.51 (0.41–0.62)	0.55 (0.42–0.73)	
25–34	0.77 (0.59–1.01)	0.68 (0.52–0.89)	
35–44	0.87 (0.66–1.15)	1.00 (0.64–1.08)	
45 or more	Ref.	Ref.	
**Education level**			0.003^*^
No education	0.26 (0.18–0.39)	0.41 (0.21–0.78)	
Primary	0.34 (0.23–0.51)	0.53 (0.29–0.99)	
Secondary	0.47 (0.31–0.70)	0.65 (0.31–1.36)	
Higher	Ref.	Ref.	
**Husband/partner education level**			0.892
No education	0.76 (0.48–1.22)	0.91 (0.29–1.39)	
Primary	0.69 (0.45–1.09)	0.93 (0.62–1.41)	
Secondary	0.77 (0.50–1.19)	0.99 (0.67–1.45)	
Higher	Ref.	Ref.	
**Wealth index**			0.323
Poorest	0.49 (0.38–0.63)	0.91 (0.62–1.34)	
Poorer	0.64 (0.51–0.79)	1.14 (0.77–1.68)	
Middle	0.72 (0.59–0.87)	1.23 (0.89–1.75)	
Richer	0.77 (0.64–0.92)	1.16 (0.85–1.59)	
Richest	Ref.	Ref.	
**Place of residence**			0.370
Rural	0.66 (0.54–0.81)	0.88 (0.67–1.16)	
Urban	Ref.	Ref.	
**Region**			0.000^**^
Southern	0.84 (0.65–1.09)	0.87 (0.65–1.18)	
Highlands	0.97 (0.75–1.27)	1.31 (1.00–1.88)	
Momase	1.43 (1.09–1.85)	1.56 (1.15–2.13)	
Islands	Ref.	Ref.	
**Religion**			0.006^*^
Christian	2.77 (1.35–5.67)	3.01 (1.38–6.59)	
Non-Christian	Ref.	Ref.	
**Own a mobile phone**			0.023^*^
Yes	1.85 (1.56–2.19)	1.29 (1.04–1.59)	
No	Ref.	Ref.	
**Read newspaper/magazine**			0.002^*^
Yes	1.12 (0.97–1.29)	1.30 (1.10–1.54)	
No	Ref.	Ref.	
**Listen to radio**			0.068
Yes	1.46 (1.23–1.73)	1.21 (0.99–1.49)	
No	Ref.	Ref.	
**Watch television**			0.408
Yes	1.43 (1.14–1.81)	0.88 (0.64–1.20)	
No	Ref.	Ref.	
**Internet access**			0.050^*^
Yes	1.79 (1.41–2.26)	1.21 (1.00–1.85)	
No	Ref.	Ref.	
**Knowledge of any contraceptive method**			0.000^**^
Yes	3.34 (2.59–4.28)	2.13 (1.58–2.87)	
No	Ref.	Ref.	
**Current type of contraceptive method used**			0.000^**^
Modern methods	1.48 (1.23–1.79)	1.26 (1.02–1.56)	
Traditional/folkloric methods	2.18 (1.73–2.75)	1.82 (1.36–2.43)	
No method	Ref.	Ref.	

* p ≤ 0.05

** p ≤ 0.001

Ref. = Reference Category; OR = Odds Ratio; CI = Confidence Interval

## Discussion

Understanding the ovulatory cycle is crucial for women to effectively plan a pregnancy or take measures to prevent it. This study investigated the determinants that influence knowledge of the ovulatory cycle among women of reproductive age in PNG. In this study, the prevalence of correct knowledge of the ovulatory cycle was reported by 21.9% of women. This finding is similar to studies conducted in Zambia (21.5%) and Rwanda (21%); however, it is much lower than findings from other African countries, such as Comoros (49%), Togo (42.8%), Sierra Leone (30.3%) and Ghana (34%) [10]. Prevalence variations in these countries may be influenced by factors such as socioeconomic status, healthcare access and utilization, and sociocultural influences. In the multivariable analysis, region, religion, mobile phone ownership, exposure to media, Internet access, knowledge about a contraceptive method, and current use of contraception were found to be significantly associated with correct knowledge of the ovulatory cycle. However, adolescent girls, young women, and those with lower education levels were less likely to have accurate knowledge of the ovulatory cycle.

In this study, women from the Highlands and the Momase regions had higher odds of correct knowledge of the ovulatory cycle compared to those in the Islands region. This could be due to collaborative efforts with relevant stakeholder partners and non-governmental organizations [28,29] that are providing essential information and mobile outreach services on sexual and reproductive health in these parts of the country. On the contrary, knowledge of the ovulatory cycle remained lower among women in the Southern region than those in other regions. This underscores the importance of providing reproductive health education and awareness, including information about ovulation, to ensure that all women have equal access to this vital knowledge, irrespective of their geographical location. PNG has several sexual and reproductive health policies, such as the *National Sexual Reproductive Policy* [30] and *Youth and Adolescent Health Policy* [31], which give significant emphasis to sexuality, reproductive health, modern contraceptives, and fertility. However, the extent to which they are implemented is unclear. Understanding of the ovulatory cycle among women in PNG is lacking, necessitating further investigation.

There is growing evidence that religious conservatism often clashes with social norms and moral values, including resistance due to differing perspectives on sexuality and contraception [32–35]. However, findings from this study revealed that women who identified as Christians were three times more likely to have correct knowledge of the ovulatory cycle than their counterparts. This could be explained by several mainline (Christian) churches that play pivotal roles in providing sexual health education in PNG, addressing the gaps where formal health services are insufficient. Most churches remain key providers of health and education services, including reproductive health, and have been leaders in the national response to HIV/AIDS in the country [28,36,37]. Their participation in reproductive health education is a possible reason for the increased understanding of the ovulatory cycle among the study participants. Furthermore, the involvement of women leaders in religious institutions, including women’s groups, may have been influential in advocating for sexual and reproductive health and addressing related issues [36]. The partnership between the church and women leaders may explain the increased understanding of the ovulatory cycle in this study. Women’s empowerment could improve the understanding of reproductive health and associated factors. However, research linking reproductive education and information and conservative views within the religious context to improve knowledge of the ovulatory cycle of women in PNG is limited.

There was a substantial association between mobile phone ownership and women’s understanding of the ovulatory cycle. Women who owned a mobile phone were more likely to have correct knowledge of their ovulation cycle. This study’s findings are similar to several studies conducted in Indonesia [38] and Kenya [39]. Furthermore, evidence suggests that women have been using mobile phone apps to map their ovulation and menstrual cycles, thereby facilitating successful conception or preventing pregnancy. For instance, an analysis of data from a self-tracking health app found a significant association between the length of the menstrual cycle, especially enabling women to predict the timing of ovulation accurately [40]. However, there is a need for more research to evaluate mobile phone users’ perspectives on reproductive technology for monitoring and understanding the ovulatory cycle in PNG.

The findings of this study showed a significant correlation between mass media exposure (i.e., newspaper or magazine, radio, and television) and women’s understanding of the ovulatory cycle. Those who read newspapers or magazines and had Internet access demonstrated higher odds of possessing correct knowledge of the ovulatory cycle compared to those without such access. This result supports studies conducted in several African countries [12], India [11], and Haiti [14] incorporating mass media in reproductive health and family planning programs. In a previous study in the Philippines and Myanmar, mass media has been an effective platform for influencing knowledge about the ovulatory cycle and contraceptive use, including the promotion of health-related behaviors [41].

Consistent with findings from Indonesia [38], knowledge about the ovulatory cycle was positively associated with mobile phone ownership and frequent Internet use. Women may have gained sufficient online information about their ovulatory cycle and specific sexual and reproductive health issues. This study suggests that women who have mobile phones and Internet access are more likely to have correct knowledge about ovulation and reproductive health. However, this study did not evaluate whether women actively use these resources for reproductive health education, indicating that access to technology does not guarantee engagement with relevant reproductive health content. Future qualitative research could examine women’s experiences with using the Internet and mobile phone technology for reproductive education and information.

Women who were knowledgeable about a contraceptive method and were using any contraceptive method had a better understanding of the ovulatory cycle than those who were not. This finding is in agreement with studies from Ethiopia [13], Haiti [14], and Malawi [42], indicating a positive association between contraceptive use and having correct knowledge of the ovulatory cycle. One reason could be that women who use modern contraception have knowledge of their ovulatory cycle and use modern contraception to avoid unintended pregnancy [14]. This could also be due to reproductive health and family planning and counseling services provided to women, which increase fertility knowledge [12]. Interestingly, those who use traditional/folkloric methods were more knowledgeable about the ovulatory cycle. While the findings acknowledge these methods, a comprehensive analysis of how cultural beliefs and gender dynamics impact understanding of the ovulatory cycle was not explored. Future qualitative research should investigate the fertility and contraceptive knowledge of women in PNG.

The findings of this study showed that women’s age was positively associated with knowledge of the ovulatory cycle. This is consistent with recent findings in sub-Saharan Africa [7,43], indicating that women of advanced maternal age are more knowledgeable of the ovulatory cycle compared to younger women. This association may indicate that as women’s age advances, their exposure to various reproductive-related issues increases, enabling them to obtain more knowledge and understanding of the topic [12]. In contrast, younger women (aged 15–24) were found to be less knowledgeable in this study. This age group, correlated with lower odds of having correct knowledge of the ovulatory cycle, was consistent with findings from similar studies [12,13,44], indicating that women at early ages of their reproductive lives have less accurate knowledge of fertility and ovulation. Poor knowledge and awareness about how age impacts fertility and preconception health underscores the need for evidence-based, tailored interventions to empower adolescents and young women to make informed reproductive choices.

Women who had no formal education or had a lower education level were less likely to have a correct understanding of the ovulatory cycle. This could be because women who are less educated are illiterate and not well-informed on reproductive health matters [14,11]. Empowering women through education and informed decision-making is key to improving reproductive health outcomes. Furthermore, empowering individuals is essential to achieving sustainable development goals, especially in ensuring universal access to sexual and reproductive healthcare [45,46]. When women are empowered and educated, they gain better awareness of their reproductive health, including fertility and contraception, enabling them to make informed decisions [12,14]. This finding highlights the need for personalized education and literacy programs on reproductive health, with special attention for those who are less educated and underprivileged.

### Strengths and limitations of the study

To the best of the authors’ knowledge, this is the first study that assessed knowledge of the ovulatory cycle and its determinants among women of reproductive age in PNG using nationally representative data. The findings are based on strong statistical power (weighted data for the sampling probabilities) and consider a complex samples analysis procedure. However, the study has limitations. It exclusively used a quantitative research design, and future qualitative studies could offer a more nuanced understanding of the factors related to correct knowledge of the ovulatory cycle among women of reproductive age. Additionally, due to the study’s cross-sectional nature, cause-effect relationships between factors and outcome variables could not be established. The DHS depends on self-reported data and is subject to social desirability and recall biases.

## Conclusion

In this study, less than a quarter of women of had correct knowledge of the ovulatory cycle. Women who were younger, identified as Christians, from the Southern region, owned mobile phones, and had access to mass media and the Internet exhibited a lack of understanding of the ovulatory cycle. This study emphasizes the need for effective strategies to improve women’s understanding of the ovulatory cycle, especially among younger, less educated, and disadvantaged. Incorporating reproductive health education into the current national education curriculum is necessary. Also, reproductive health promotion and awareness through mass media and communication platforms can effectively enhance women’s knowledge of the ovulatory cycle and empower them to make informed decisions about their reproductive health.

## Supporting information

S1 Table Variance Inflation Factor (VIF). (DOCX)

S2 Table The STROBE guidelines for cross-sectional studies. (DOCX)
