# Supplementary material for: Knowledge of the ovulatory cycle and its determinants among women of reproductive age in Papua New Guinea: Insights from a population-based study
Source: PLoS One. 2025 May 28;20(5):e0324255. doi: 10.1371/journal.pone.0324255 (PMC12118850; doi:10.1371/journal.pone.0324255)
Supplement: S1 Table — (DOCX) [file pone.0324255.s001.docx]

S1 Table: Variance Inflation factor (VIF)

| **Variables** | **VIF** | **1/VIF** |
| --- | --- | --- |
| Mean VIF | 1.29 |  |
| Age (years) | 1.13 | 0.88 |
| Education level | 1.66 | 0.60 |
| Husband/partner education level | 1.06 | 0.94 |
| Respondent currently working | 1.04 | 0.97 |
| Wealth index | 1.83 | 0.55 |
| Place of residence | 1.31 | 0.76 |
| Region | 1.04 | 0.96 |
| Religion | 1.01 | 0.99 |
| Owns a mobile phone | 1.42 | 0.70 |
| Read newspaper/magazine | 1.03 | 0.97 |
| Listen to radio | 1.56 | 0.64 |
| Watch television | 1.69 | 0.59 |
| Internet access | 1.52 | 0.66 |
| Knowledge of any contraceptive method | 1.20 | 0.83 |
| The current contraceptive method used by type | 1.12 | 0.89 |
| Recent sexual activity (last 4 weeks) | 1.00 | 0.99 |
